# Treatment of pre- and confirmed cervical cancer in HIV-seropositive women from developing countries: a systematic review

**DOI:** 10.1186/s13643-020-01345-2

**Published:** 2020-04-10

**Authors:** Witness Mapanga, Elvira Singh, Shingairai A. Feresu, Brendan Girdler-Brown

**Affiliations:** 1 School of Health Systems and Public Health, Epidemiology & Biostatistics, University of Pretoria, 5-10 H.W. Snyman Building, Pretoria, South Africa; 2 Non-Communicable Diseases Research (NCDR) Division of the Wits Health Consortium, Faculty of Health Sciences, University of the Witwatersrand, Johannesburg, South Africa; 3 Brooklyn, South Africa; 4 Cancer Epidemiology Research Group, National Cancer Registry, National Health Laboratory Service, Johannesburg, South Africa; 5 Community Medicine Unit, School of Public Health, University of the Witwatersrand, Johannesburg, South Africa; 6 Faculty of Health Sciences, The University of Fort Hare, P.O. Box 1054, 45 Church Street, Gasson Building, 7th Floor, East London, 5201 South Africa

**Keywords:** Cervical neoplasia, Cervical cancer, Treatment, HIV, Developing countries

## Abstract

**Background:**

Cervical cancer has become a major public health challenge in developing countries with a reported age-standardised incidence rate of about 17.9/100,000/year and lifetime risks approaching 1 in 20 in some settings. Evidence indicates that HIV-seropositive women are 2 to 12 times more likely to develop precancerous lesions that lead to cervical cancer than HIV-negative women. There is a lack of rigorous evidence on which treatment methods are being utilised for HIV-positive women, and this review aims to synthesise available evidence on treatment modalities for both cervical neoplasia and cervical cancer in HIV-seropositive women in developing countries.

**Methods:**

A systematic review guided by a published protocol was conducted. Online databases including MEDLINE/PubMed, Embase, CINAHL and Emerald (via EBSCOhost), PsycINFO, Cochrane Library, and health databases, which cover developing countries (3ie Systematic Reviews, WHO library and databases, World Bank website), were searched for published articles. Additional articles were found through citation, reference list tracking, and grey literature. Study design, treatment category, geographic country/region, and key outcomes for each included article were documented and summarised.

**Results:**

Thirteen research articles from sub-Saharan Africa, Asia, and South America were included. Eight (61.5%) articles focused on the treatment of cervical cancer with the remaining five (38.5%) assessed cervical neoplasia treatment. The available cervical cancer treatments, radiotherapy, chemotherapy, chemoradiation, and surgery are effective for HIV-seropositive patients, and these are the same treatments for HIV-negative patients. Both cryotherapy and LEEP are effective in reducing CIN2+ among HIV-seropositive women, and a choice between the treatments might be based on available resources and expertise. Radiation, chemotherapy, concurrent treatment using radiotherapy and chemotherapy, and surgery have shown the possibility of effectiveness among HIV-seropositive women. Cervical cancer stage, immunosuppressive level including those on HAART, and multisystem toxicities due to treatment are associated with treatment completion, prognostic, and survival outcomes.

**Conclusions:**

Treatment of cervical cancer is based on the stage of cancer, and poor outcomes in most developing countries might be due to a lack of optimal treatment regimen. Those infected with HIV were younger and had advanced cervical cancer as compared to those who were HIV-negative. Facilitation and putting HIV-infected people on life-long ART is of importance and has been found to have a positive impact on cervical cancer treatment response. Research on precancerous lesions and cervical cancer management of HIV-seropositive patients focusing on the quality of life of those treated; the effectiveness of the treatment method considering CD4+ count and ART is required.

**Systematic review registration:**

PROSPERO CRD42018095707

## Background

Persistent infection with human papillomavirus (HPV) is the main risk factor for developing cervical cancer [1–4]. The HIV burden has seen an increase in morbidity and mortality due to cervical cancer, especially in developing countries, where HIV incidence and prevalence are high [5–7]. In these developing countries, the reported age-standardised incidence rate can be as high as 17.9/100,000/year [8]. Also, most of these patients are mostly diagnosed at an advanced stage due to a lack of coordinated and systematic screening [9, 10]. The management of both cervical neoplasia and cervical cancer in HIV-seropositive women, who mostly reside in developing countries, is bound to be challenging especially with the potential risk of medical complications, and resource constraints including unavailability of treatments [7]. There are several treatment modalities for cervical cancer, namely, chemotherapy, surgical management, and radiation therapy [7, 11, 12]. Treatment modalities for precancerous lesions and cervical cancer are based on the stage of the lesions and available resources; the associated poor outcomes of treatment among HIV seropositive women in developing countries may be due to lack of optimal treatment regimen [10, 13]. Most developing countries lack skilled surgeons to carry out radical surgery for cervical cancer, and this has left HIV-seropositive cervical cancer patients with few treatment options. In cases where surgeons are available, surgery is expensive and out of reach of many, who happen to be poor [14]. In developing countries especially sub-Sahara Africa, many women with cervical cancer have no access to radiotherapy, further limiting their treatment options. However, little or no information exists that has shown that any of the current treatments are effective compared to other treatments when it comes to treating cervical cancer in HIV-seropositive women. In sub-Saharan Africa, treatments like radiation therapy and other surgical procedures are not fully utilised because of lack of equipment and qualified personnel; hence, little has been documented on which treatment procedures are being used for the treatment of cervical cancer in HIV seropositive women [15]. Therefore, there is a lack of evidence-based guidelines and strategies for the treatment of cervical cancer in HIV-seropositive women in most developing countries. Coupled with this, there is little rigorous evidence on the global epidemiology of the treatment of cervical cancer in HIV-seropositive women [15]. This review aims to answer the following questions: What are the treatment modalities that are being used to treat and manage cervical cancer in HIV-seropositive women in developing countries? Are these the same treatment modalities that are being used for HIV-negative women? Are the treatment modalities effective in HIV-seropositive women? This review will investigate and identify the existing treatment modalities used for cervical cancer in HIV-seropositive women in some of the developing countries.

## Methods

### Search strategy

**Fig. 1:**
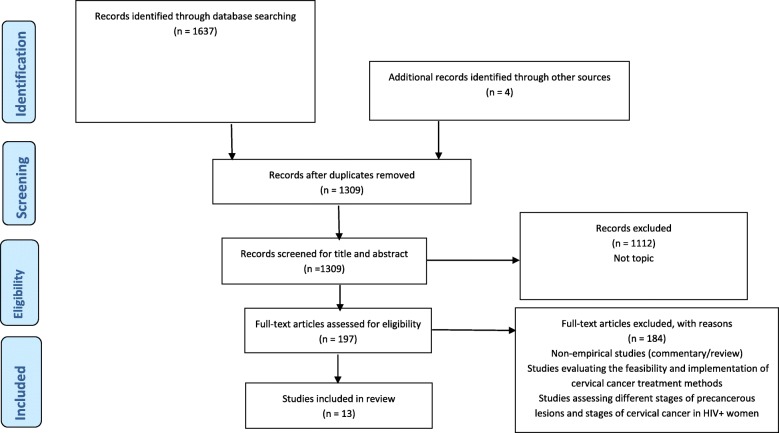
PRIMSA flowchart. The search strategy is reported according to PRISMA guidelines

This review was guided by a protocol [16] and registered with PROSPERO (CRD42018095707). The PRISMA guidelines (Fig. 1) informed the reporting of this systematic [17]. Two independently working reviewers (WM and SF) searched MEDLINE/PubMed (1966 to present, using Medical Subject Heading (MeSH) terms), Embase (1980 to present) via the OVID interface, and Cochrane, PsycINFO, Emerald, and CINAHL (all from 1961 to present) via EBSCOhost, using a combination of the following words: “Cervical Neoplasm”, “Cervical Cancer”, “treatment”, and “HIV”. The initial results (2753 records) were further narrowed down by adding “developing countries” (individual database search strategies are included in Additional file 1). In addition, the two reviewers (WM and SF) also searched the 3ie Systematic Reviews, WHO library and databases, World Bank website and WHO ICTRP and cliniccaltrials.gov [16] and identified an additional 4 studies. All the searches included articles published up to 31 January 2020, which was also the last date of the searches.

### Eligibility criteria

Studies were eligible for inclusion if they investigated cervical neoplasia or cervical cancer treatment methods (cryotherapy, loop electrosurgical excision procedure (LEEP), chemotherapy, surgery, and radiation therapy among others) for HIV-positive women in developing countries, peer-reviewed, grey literature (dissertations, conference papers, government reports), and done for or in countries or regions considered developing by the United Nations [16]. Studies were excluded if they had unrepresentative samples.

### Study selection

The search of databases and grey literature yielded 1637 results, and an additional four studies were identified through reference tracking to make a total of 1641 articles (Fig. 1). All the articles were combined into EndNote reference management software, and 332 duplicates were removed. The remaining 1309 articles were exported to Covidence software, where duplicate screening was performed. Two independently working reviewers (WM and SF) conducted title and abstract screening based on the relevance to the review question. Studies were excluded when the title and abstract mentioned cervical cancer screening or vaccination or described the implementation process of cervical cancer treatment. Disagreements related to the screening process were resolved as a team through discussions as reported in the published protocol [16]. Through title and abstract screening, 1112 articles were excluded. Two independent reviewers (WM and BGB) conducted full-text screening on the remaining 197 articles, and 184 articles did not meet the eligibility criteria and were excluded. A total of 13 articles met the eligibility criteria and included in the final analysis (Fig. 1).

### Data extraction

Two independent reviewers (WM and SF) conducted double data extraction in Covidence software on the 13 articles that were included in the final analysis, whilst the rest of the team checked for quality and consistency. A data extraction form, as indicated in the published protocol [16], guided the data extraction process. The quality of the data extraction form was determined by piloting 5 articles and the team discussing and resolving all the inconsistencies through consensus. The following variables were extracted from the studies: first author and publication year, the title of the study, study type, aim of the study, participants and their age, study setting, stage of cervical cancer, treatment method, outcomes, results, and authors’ conclusions.

### Quality assessment of included studies

For quality assessment of the studies, the team utilised a combination of the NIH Study Quality Assessment Tools for observational cohort and cross-sectional, case-control, and before-after studies, together with a modified version of the Newcastle-Ottawa Quality Assessment Scale [18, 19]. For the quality assessment process to be easy to conduct, studies were grouped into observational studies without a control group(s), observational studies with a control group(s), and randomised controlled trials [16]. The following factors were considered of importance during the quality assessment process: a clearly defined and specified study population, clearly defined and valid exposure and outcomes measures, adequate sample sizes, randomisation of participants, and a prespecified inclusion and exclusion criteria for being in the study. Two independent reviewers (WM and SF) conducted the quality assessment, and disagreements during the process were resolved through discussion by all the four authors. Answers to the questions of the two checklists gave an overall score of each article. Each paper was scored to a maximum of 12 points, with every ‘yes’ answer carrying 1 mark and ‘no’ answer carrying zero marks. An average of the scores from the two reviewers became the final quality score for each study. Quality was benchmarked as low if the average score of the two assessors was between 0 and 4, moderate if the average score was from 5 to 8, and high if average score was from 9 to 12. However, no studies were excluded based on quality.

## Results

### Study selection and characteristics of the included studies

**Fig. 2:**
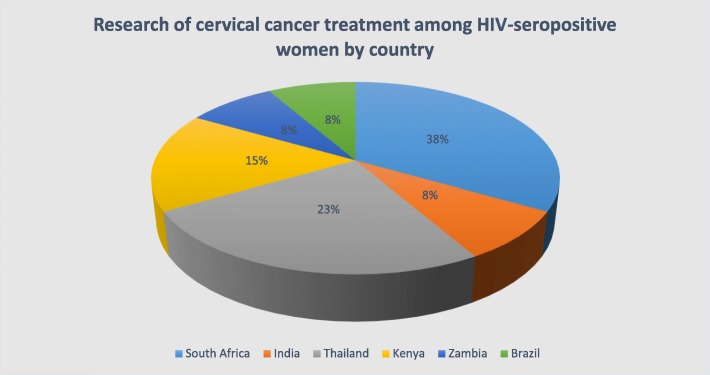
Research of cervical cancer treatment among HIV-seropositive women by country

Out of the initial 1309 (after 332 duplicates were removed), 13 studies (of over 2800 patients) met the inclusion criteria and were included to form the basis of the analysis. Most of the studies were excluded in the title and abstract screening stage because they were not relevant to the topic under review. Under full-text screening, studies were excluded because they were non-empirical, evaluating the feasibility and implementation of cervical cancer treatment methods, and assessing different stages of precancerous lesions and stages of cervical cancer in HIV-positive women. Most of the included studies (69.2%) were published after the year 2010. The studies represented three regions, sub-Saharan Africa 8 (61.5%), Asia 4 (30.8%), and South America 1 (7.7%), as indicated in Fig. 2.

The 13 included studies evaluated, assessed, or compared the effectiveness, treatment response, and outcomes of different cervical neoplasia and cervical cancer treatment methods for HIV-seropositive women. The results are presented in themes, that is, treatment methods of cervical neoplasia first, followed by treatment methods of confirmed cervical cancer. Five of the included studies (38.5%) are prospective cohort, evaluating treatment response and toxicity to a combination of radiotherapy and chemotherapy, treatment with surgery and radiation, and treatment with loop electrosurgical excision procedure (LEEP). Four (30.8%) retrospective cohort studies and one (7.7%) evaluated the survival outcomes of chemotherapy, treatment outcomes of radiotherapy, and complications with LEEP, and compared clinical characteristics after radiation and chemotherapy. Two (15.4%) randomised controlled trials compared the efficacy of LEEP vs cryotherapy and cryotherapy with no treatment. One (7.7%) case study examined the results of a radical hysterectomy surgery on two different patients. All the 13 studies were almost consistent in defining their outcomes, such as treatment response, clinical/prognostic characteristics, survival response, and mortality rates. However, baseline characteristics of participants included in the studies were different, with age ranging from 18 years old to well above 55 years old. Sampling and recruitment of the participants were also different. In addition, participants had different stages of both precancerous lesions and cervical cancer, some were on highly active antiretroviral therapy (HAART), whilst others were not on HIV treatment, and the follow-up intervals were different as well (see Table 1).

**Table 1:** Table of evidence

First author & publication year	Study type	Purpose	Population & age	Country	Stage of cancer	Treatment method	Outcome(s)	Results	Authors’ Conclusions	Quality score
**Simonds et al.,** **2012** [20]	Retrospective cohort study	To compare the clinical characteristics, radiation, and chemotherapy treatments, outcomes in a cohort of HIV-positive and HIV-negative women with cervical cancer	59 HIV-positive (median age 41 years) and 324 HIV-negative (median age of 50 years) patients	South Africa	IBi–IIIB	Radiation and chemotherapy	Chemotherapy cycles, response at time of brachytherapy, and 6-week follow-up	88.1% of HIV-positive patients presented with IIIB disease compared to 65.7% of HIV-negative patients (*p* = 0.009). 79.7% HIV-positive and 89.8% HIV-negative patients completed radiation dose of 68 Gy EBRT and HDR brachytherapy (*p* = 0.03). For concurrent chemotherapy, 53.1% HIV-positive and 74.6% HIV-negative patients completed four or more weekly cycles of platinum-based treatment. At 6 weeks, poor response was associated with stage IIIB disease (OR = 2.39, 95% CI 1.45–3.96) and receiving less than 68 Gy EQD_2_ radiation (OR = 3.14, 95% CI 1.24–794).	Good medical care of HIV-positive patients can enable patients to complete treatment for locally advanced cervical cancer and might improve response to treatment.	Moderate
**Shrivastava et al., 2005** [21]	Retrospective review	To determine the effect of radiotherapy in HIV seropositive cervical cancer patients, tumour response and toxicity, and compliance of patients to the treatment.	42 HIV seropositive patients, mean age of 41 years	India	IIIB–IVA	Radiotherapy	Age and symptoms of presentation, clinical stage, response, compliance, and tolerance to radiotherapy	All patients presented with the symptoms of cervical disease. Of these patients 31 (74%) patients had ‘Karnofsky Performance Scale’ (KPS) more than 80%. Twenty-one (50%) of the patients were of stage IIIb–IVa. Thirty-two (76%) were started on radiotherapy with radical intent. Compliance to radiotherapy was poor with 24% patients discontinuing after few fractions of radiotherapy. Seven (17%) patients were given palliative radiotherapy. Twenty-two patients completed prescribed radical radiotherapy and 50% of these achieved complete response. Grade III–IV acute gastrointestinal toxicity was seen in 14% of the patients, and grade III acute skin toxicity was seen in 27% of patients, leading to treatment delays. There was good relief of symptoms in patients treated with palliative intent.	Radiotherapy is effective in this set of patients. Palliative fractionation schedules are effective for patients with poor performance status and locally advanced cancers in relieving the symptoms related to carcinoma cervix. An emphasis should be given to the increased acute mucosal and skin toxicity and to improving compliance and clinical outcome of these patients.	Low
**Gichangi et al., 2006** [22]	Prospective cohort study	To determine the impact of HIV infection on acute morbidity and pelvic tumour control following external beam radiotherapy (EBRT) for cervical cancer	218 patients, 20% of them HIV-positive	Kenya		Radiotherapy	Acute treatment toxicity and pelvic tumour control	Overall, 53.4% of the patients had radiation-related acute toxicity (grade 3–4). HIV infection was associated with a 7-fold higher risk of multisystem toxicity: skin, gastrointestinal tract (GIT), and genitourinary tract (GUT) systems. It was also an independent risk factor for treatment interruptions (adjusted relative risk 2.2). About 19% of the patients had residual tumour at 4 and 7 months post-EBRT. HIV infection was independently and significantly associated with 6-fold higher risk of residual tumour post-EBRT. The hazard ratio of having residual tumour after initial EBRT was 3.1-times larger for HIV-positive than for HIV-negative patients (*p* = 0.014).	HIV is associated with increased risk of multisystem radiation-related toxicity; treatment interruptions and pelvic failure (residual tumour) following EBRT. HIV infection is an adverse prognostic factor for outcome of cervical cancer treatment.	Moderate
**Mdletshe et al., 2016** [23]	Prospective quantitative comparative study	To evaluate in detail treatment response, its toxicities and compliance of HIV-positive women to radical combination therapy (radiotherapy with chemotherapy)	55 HIV-positive (median age of 40 years) and 55 HIV-negative (median age of 55 years) patients with performance status ECOG I & II	Zambia	IB_2_–IIIB	Combination of radiotherapy and chemotherapy given concurrently	Acute reactions to radical chemo-radiation and toxicity	All participants completed EBR and HDR as prescribed. Average EBR dose delivered was 48 Gy, and the difference in dose received was significant with regard to HIV status (*p* = 0.022). 58% of HIV-positive were treated with 6.5 Gy × 4 brachytherapy fractions as compared to 58% HIV-negative patients treated with 8 Gy × 3 fractions. There were no statistically significant differences in toxicity between HIV-positive and HIV-negative patients with regard to skin, GIT system, GU system, and haemopoietic system.	Radical chemo-radiation in conventional doses was safely tolerated by a well-selected cervical cancer HIV-positive group on HAART and could be considered suitable for similar patients.	Moderate
**Boupaijit and Suprasert, 2016** [24]	Retrospective study	To evaluate the survival outcomes of chemotherapy and the prognostic factors in this setting	173 patients (mean age of 50.9 year), with 4.1% of them HIV-positive	Thailand	IVB	Chemotherapy	Survival outcomes and prognostic factors	Median overall survival of all studied patients was 13.2 months. Only a recurrence-free interval of less than 12 months was an independent prognostic factor for survival outcome	Chemotherapy treatment for advanced and recurrent cervical cancer patients showed modest efficacy with a shorter recurrence-free survival less than 12 months as a significant poor prognosis factor	Moderate
**Ferreira et al., 2017** [25]	Cohort study	To assess mortality, treatment response, and relapse among HIV-infected and HIV-uninfected women with cervical cancer in Rio de Janeiro, Brazil	87 HIV-infected and 336 HIV-uninfected women with cervical cancer	Brazil	IA/IB1, IB2/II, III, IVA/IVB	28% treated with surgery, 23% with radiation, 30% with chemo-radiation, and 36% received additional brachytherapy	Mortality, treatment response and relapse	70% HIV-infected women and 76% HIV-uninfected women completed recommended treatment. 58 HIV-infected and 176 HIV-uninfected women died. Among HIV-infected women, overall mortality was 324 per 1000 person-years, with 82% of deaths due to cancer. Among HIV-uninfected women, overall mortality was 209 per 1000 person-years, with 93% of deaths from cancer. Among 222 patients treated with radiotherapy, HIV-infected had similar response rates to initial cancer therapy as HIV-uninfected women (HR 0.98, 95% CI 0.58–1.66). However, among women who were treated and had a complete response, HIV was associated with elevated risk of subsequent relapse (HR 3.60, 95% CI 1.86–6.98, adjusted for clinical stage)	HIV infection was not associated with initial treatment response or early mortality, but relapse after attaining a complete response and late mortality were increased in those with HIV. There is a role for an intact immune system in control of residual tumour burden among treated cervical cancer patients	Moderate
**Moodley, 2017** [26]	Case studies	To present radical hysterectomy experience to inform management of early-stage invasive cervical cancer	18-year-old nulliparous, 36-year-old primiparous, and 39-year-old para 2 HIV-positive women	South Africa	Differentiated squamous cell carcinoma (LVSI)	Surgery (radical hysterectomy)	Management outcomes after radical hysterectomy	All three made uneventful postoperative recoveries and all vaginal vault cytologic smears have been negative. The 18-year-old is well 6 years postsurgery as are the 36- and 39-year-olds at 3 years follow-up visits. The 39-year-old patient needed ureteric re-implantation due to ureteric stricture, which could occur as a recognised complication even in HIV Non-infected patients.	With reasonable levels of immunosuppression, management of HIV-positive women with early cervical cancer with radical hysterectomy can produce reasonable outcomes and survival.	Low
**Kietpeerakool et al. 2006** [27]	Retrospective cohort study	To evaluate the treatment outcomes and complications in human immunodeficiency virus (HIV)-infected women undergoing loop electrosurgical excision procedure (LEEP) for cervical neoplasia	60 HIV-infected (mean age of 35.9 years) and 61 HIV-negative (mean age of 40.1 years) women with cervical neoplasia	Thailand	LSIL–HSIL	LEEP	LEEP treatment outcomes and complications in HIV-positive women	97.1% and 88% of HIV-positive women were disease-free at 6 and 12 months, respectively after LEEP. 1.7% had severe intraoperative haemorrhage, 5% had early and late postoperative haemorrhage, 11.7% had localised infection of the cervix and 3.3% developed cervical stenosis at 6 months after LEEP. No significant difference in overall complications (*p* = 0.24) between HIV-positive and HIV-negative patients.	LEEP appears to be safe and effective in HIV-infected women.	Moderate
**Firnhaber et al. 2017** [28]	Randomised controlled trial	To compare cervical cryotherapy to observation in HIV-infected women with CIN1 on histology	202 HIV-positive women (median age of 37.9 years) with CIN1	South Africa	CIN1	Cryotherapy	CIN2/3 by histology at month 12. Regression of cervical histology to no evidence of NILM	CIN2/3 at month 12, occurred in 2 of 99 (2%) women in the cryotherapy arm as compared with 15 of 103 (15%) women in the no treatment arm [86% risk reduction, 95% confidence interval (CI) 61 to 97%; *p* = 0.0016]. No cervical cancers in both arms. Forty of 99 (40%) women in the cryotherapy group experienced regression as compared 14 of 103 (14%) women in the no treatment group (69% reduced regression, 95% CI 58% to 83%, *p*<0.0001).	Treating CIN1 with cryotherapy reduces progression to CIN2/3. The benefit was exclusively among those with hrHPV. Cryotherapy was safe in this population with no serious adverse events.	High
**Woo et al., 2011** [29]	Prospective cohort study	To estimate the safety, tolerability, and acceptability of loop electrosurgical excision procedure (LEEP) for cervical intraepithelial neoplasia (CIN 2/3) in HIV-positive women	180 HIV-positive women	Kenya	CIN2/3	LEEP	Safety, tolerability and acceptability of LEEP after 4 weeks post-procedure	179 (99%) reported “very mild” to mild symptoms, while 1 (*n* = 1%) participant described the symptoms as moderate. Mean CD4+ count was significantly higher among women who reported any symptoms compared to women who reported no symptoms post LEEP (419 cells/mm^3^ vs. 349 cells/mm^3^, *p* < 0.05) Only 16% (CI 11–22%, *n* = 29) of women reported early resumption of intercourse prior to their 4-week follow-up visit	LEEP performed by clinical officers was well-accepted by HIV positive women and appears safe, resulting in minimal side effects, even among women with early resumption of intercourse	Moderate
**Kietpeerakool et al., 2006** [27]	Prospective study	To assess outcome in HIV-positive women undergoing the loop electrosurgical excision procedure (LEEP)	70 HIV-positive (mean age of 37.5) and 719 HIV-negative (mean age 45.8) women.	Thailand	CIN1/2/3, IA1-IB1	LEEP	Safety of LEEP among HIV-positive patients	HIV infection was not significantly associated with the incidence of LEEP complications (adjusted odds ratio, 0.41; 95% CI, 0.15–1.15; *p* = 0.10). There were no statistically significant differences in operative time, size of excised specimens, incidence of 2 or more passes of the loop, or use of Monsel paste between the 2 groups. There was a higher prevalence of LEEP margin involvement in the HIV-positive than in the HIV-negative group (60.0% vs 49.4%).	LEEP is safe in HIV-infected women with cervical neoplasia treated in outpatient settings, and when technically possible, a repeat intervention is safe, with an acceptable success rate, even though HIV-infected women have a higher risk of resection margin involvement.	Moderate
**Einstein et al., 2019** [30]	Randomised controlled	To determine the feasibility, safety, and tolerability of concomitant chemoradiotherapy administered at standard doses in HIV-infected women with locally advanced cervical cancer (LACC) receiving antiretroviral therapy (ART).	38 HIV-seropositive women over 18 years old	Sub-Saharan Africa	LACC	Concomitant chemoradiotherapy	Feasibility, safety, and tolerability of concomitant chemoradiotherapy administered at standard doses	Sixty-four women were screened at two sites in sub-Saharan Africa, of whom 40 eligible participants were enrolled, for a screening ratio of 1.60. Of the 38 eligible participants who initiated study treatment, 31 (82%) completed treatment. By the 12-month follow-up visit, 7 women had died of disease and 29 of 31 (94%) returned for follow-up. One-year progression-free survival was 76.3% (95% CI, 59.4–86.9%), and did not significantly differ according to stage at entry (*p* = 0.581). Participant-reported adherence to ART was high; by 12 months, 93% of participants had an undetectable viral load. The most common grade 3 or 4 adverse event was decreased lymphocyte count that affected all treated participants. Non-hematologic serious adverse events were similar to those observed in women with LACC without HIV infection.	The majority of HIV-infected women with LACC can complete concomitant chemoradiotherapy with the same cisplatin dose used in HIV-uninfected women with comparable tolerability and high ART adherence while on treatment.	High
**Smith et al., 2017** [31]	Randomised controlled trial	To identify effective treatment methods for high-grade cervical precursors among HIV-seropositive women by comparing the difference in the efficacy of loop electrosurgical excision procedure vs cryotherapy for the treatment of high-grade cervical intraepithelial neoplasia (grade ≥ 2)	166 HIV-seropositive women aged 18–65 years	South Africa	CIN2+	Cryotherapy vs LEEP	Efficacy of LEEP and cryotherapy	Cumulative cervical intraepithelial neoplasia grade ≥ 2 incidence was higher for cryotherapy (24.3%; 95% confidence interval, 16.1–35.8) than LEEP at 6 months (10.8%; 95% confidence interval, 5.7–19.8) (*p* = .02), although by 12 months, the difference was not significant (27.2%; 95% confidence interval, 18.5–38.9 vs 18.5%; 95% confidence interval, 11.6–28.8, *p* = .21). Cumulative cervical intraepithelial neoplasia grade ≥ 1 incidence for cryotherapy (89.2%; 95% confidence interval, 80.9–94.9) did not differ from LEEP (78.3%; 95% confidence interval, 68.9–86.4) at 6 months (*p* = .06); cumulative cervical intraepithelial neoplasia grade ≥ 1 incidence by 12 months was higher for cryotherapy (98.5%; 95% confidence interval, 92.7–99.8) than LEEP (89.8%; 95% confidence interval, 82.1–95.2) (*p* = .02). Cumulative high-grade cytology incidence was higher for cryotherapy (41.9%) than LEEP at 6 months (18.1%, *p* < .01) and 12 months (44.8% vs 19.4%, *p* < .001). Cumulative incidence of low-grade cytology or greater in cryotherapy (90.5%) did not differ from LEEP at 6 months (80.7%, *p* = .08); by 12 months, cumulative incidence of low-grade cytology or greater was higher in cryotherapy (100%) than LEEP (94.8%, *p* = .03).	Both treatments appeared effective in reducing cervical intraepithelial neoplasia grade ≥ 2 by > 70% by 12 months. The difference in cumulative cervical intraepithelial neoplasia grade ≥ 2 incidence between the 2 treatment methods by 12 months was not statistically significant. Relatively high cervical intraepithelial neoplasia grade ≥ 2 recurrence rates, indicating treatment failure, were observed in both treatment arms by 12 months. A different treatment protocol should be considered to optimally treat cervical intraepithelial neoplasia grade ≥ 2 in HIV-seropositive women	High

### Treatment options for cervical neoplasia for HIV seropositive women

Five (38.5%) of the 13 included studies evaluated efficacy, treatment outcomes, and complications in HIV-seropositive women with cervical neoplasia treated with LEEP or cryotherapy. Three studies evaluated LEEP [27, 29, 32], one compared cryotherapy with no treatment [28], and the other compared LEEP and cryotherapy to identify effective treatment [31].

#### LEEP

Three studies reviewing LEEP among HIV-positive women concluded that the procedure is safe and effective. A retrospective cohort study in Thailand evaluated treatment outcomes and complications of HIV-infected and HIV-negative women with a low-grade squamous intraepithelial lesion (LSIL) or high-grade squamous intraepithelial lesions (HSIL) undergoing LEEP [27]. The HIV-infected cohort had a mean age of 35.9 years as compared to 40.1 years of the HIV-negative cohort. After 6 and 12 months of LEEP, 97.1% and 88.0% of HIV-infected women had no cervical neoplasia, respectively. In terms of complications, there was no significant difference (*p* = 0.24) when compared to HIV-negative women [27]. These findings were almost similar to evidence generated in the same country 2 years later, which found out that there was no significant association between HIV and LEEP complications among women with cervical intraepithelial neoplasia (CIN) grades 1, 2, 3, and cervical cancer stage 1A1–1B1 [32]. In Kenya, a prospective cohort study also confirmed that LEEP was well tolerated and accepted by HIV-positive women who had CIN 2 and 3, with 99.0% of participants reporting ‘very mild’ symptoms of complications. Also, women with a higher mean CD4+ count were likely to report symptoms of complications as compared to women with lower mean CD4+ counts [29].

#### Cryotherapy

In a randomised controlled trial in South Africa among HIV-infected women with CIN1, treatment with cryotherapy was found to significantly reduce progression to CIN2/3. After 12 months, only 2% of women undergoing cryotherapy treatment as compared to 15% not receiving treatment (86% risk reduction, 95% CI 69–97%, *p* = 0.0016) progressed to CIN2/3. Regression was also significant in women receiving cryotherapy as compared to those not receiving treatment (69% reduced regression, 95% CI 58–83%, *p* = 0.0001) [28].

#### Cryotherapy vs. LEEP

To try and identify an effective treatment method between cryotherapy and LEEP for high-grade cervical precursors (CIN2+) among HIV-seropositive women, a randomised controlled trial was conducted in South Africa [31]. After 6 months of treatment, there was a higher cumulative CIN2+ incidence for cryotherapy (24.3%, 95% CI 16.1–35.8) as compared to LEEP (10.8%, 95% CI 5.7–19.8) at *p* = 0.02. However, after 12 months of treatment, there was no significant difference between the two (27.2%, 95% CI 18.5–38.9 vs. 18.5%, 95% CI 11.6–28.8) at *p* = 0.21 [31]. Both cryotherapy and LEEP are effective in reducing CIN2+, and a choice might be based on available resources and expertise.

### Treatment options for cervical cancer for HIV seropositive women

Treatment of cervical cancer with radiation, chemotherapy, concurrent treatment using radiotherapy and chemotherapy, and surgery among HIV-seropositive women was evaluated in 8 (61.5%) out of the 13 included studies. The results of the treatment options are reported as themes as follows.

#### Chemotherapy

A retrospective study in Thailand on 173 HIV-positive and HIV-negative patients (with a mean age of 50.9 years) with stage IVB cervical cancer showed modest efficacy, with overall median survival among all patients of 13.2 months. The only independent prognostic survival outcome was a recurrence-free interval of fewer than 12 months [24]. In Brazil, HIV was found not to be associated with mortality due to cervical cancer during the first year post-treatment, but the association was significant after more than 1 to 2 years post-diagnosis (overall mortality: adj HR = 2.02; 95% CI 1.27–3.22; cancer-specific mortality 4.35, 1.86–10.2) [25].

#### Radiotherapy

A retrospective review conducted in India to determine radiotherapy’s effect on HIV-seropositive women of mean age of 41 years with cervical cancer stage IIIB–IVA indicated that radiotherapy is effective, but compliance to the treatment is poor (with only 52.4% of women completing the prescribed radical radiotherapy and 50.0% of them achieving complete response) [21]. To overcome poor compliance, palliative radiotherapy schedules were prescribed, and these were identified to be effective for HIV-seropositive women with cervical cancer [21]. Despite it being effective, evidence has shown that those undergoing radiotherapy present with acute skin toxicity (grade III) and grade III–IV acute gastrointestinal toxicity [21]. These findings were supported by a prospective cohort study conducted in Kenya, which showed that there was a 7-fold higher risk of developing multisystem (skin, gastrointestinal, and genitourinary) toxicity if HIV-infected and have undergone through radiotherapy [22]. This multisystem toxicity was found as a factor contributing to the interruption of treatment (adj. RR = 2.2) [22]. Follow-ups at 4- and 7-months post-radiotherapy indicate that HIV-seropositive is 6-fold at risk of having a residual tumour (HR = 3.1, *p* = 0.0014) as compared to patients who are HIV-negative [22]. This finding was in accord with what was suggested in Brazil where there was an elevated risk of subsequent relapse for HIV-seropositive women as compared to HIV-negative women (HR = 3.60; 95% CI 1.86–6.98) [25].

#### Radiation and chemotherapy

To compare the clinical characteristic outcomes after radiation and chemotherapy among HIV-positive (median age of 41 years) and HIV-negative women (median age of 50 years) with cancer stage IBi-IIIB, a retrospective cohort study was conducted in South Africa [20]. Treatment completion rates between the two patient cohorts were different, with 79.7% of HIV-positive and 89.8% HIV-negative completing their radiation dose and brachytherapy (*p* = 0.03). For concurrent chemotherapy, only 53.1% HIV-positive and 74.6% HIV-negative managed to complete 4 or more weekly cycles. After 6 weeks, poor response to treatment was significantly associated with stage IIIB (OR = 2.39, 95% CI 1.45–3.96) and receiving less than recommended radiation dose (OR = 3.14, 95% CI 1.24–7.94) [20].

#### Combination of radiotherapy and chemotherapy

A prospective quantitative comparative study in Zambia evaluated the treatment response, treatment toxicities, and compliance to radical chemo-radiation among both HIV-positive (median age of 40 years) and HIV-negative (median age of 55 years) women with stage IB_2_-IIIB cancer [23]. As opposed to a failure to complete treatment as indicated by evidence in South Africa [20], all participants in this prospective study completed their treatments. Well-selected HIV-positive cervical cancer patients on HAART can safely tolerate radical chemo-radiation in conventional doses [23]. The difference in chemo-radiation doses (6.5 Gy × 4 for 58% of HIV-positive women vs. 8Gy × 3 for 58% of HIV-negative women) was significant to HIV status (*p* = 0.022). In terms of toxicity (regarding GIT system, skin, haemopoietic system, and GU system), there were no significant differences between HIV-positive and HIV-negative patients [23]. In a study of 38 HIV-positive women with locally advanced cervical cancer, the safety, tolerability, and feasibility of concomitant chemoradiotherapy were assessed in two sites in sub-Saharan Africa. Results indicated that HIV-infected women (82%) who adhere to ART can tolerate and complete concomitant chemoradiotherapy as HIV-negative women. After 1 year with 7 women dead due to cervical cancer, 29 of the remaining 31 (94%) returned for a scheduled clinical visit and progression-free survival was at 76.3% (95% CI, 59.4–86.9%) [30].

#### Surgery (radical hysterectomy)

Three case studies in South Africa of HIV-positive women with LVSI, an 18-year-old nulliparous, 36-year-old primiparous, and 39-year-old para-2, examined the radical hysterectomy to inform management of early-stage invasive cancer [26]. After 6 years post-surgery, the 18-year old has recovered, and all the vaginal vault cytologic smears have come negative. At 3 years of follow-up visits, both the 36- and 39-year olds have also recovered and with negative vaginal vault cytologic smears [26].

## Quality assessment of included studies

Few studies (*n* = 2, 15.4%) were determined to be of ‘high’ quality using a combination of the modified Newcastle-Ottawa Quality Assessment Scale and the NIH Study Quality Assessment Tools for observational cohort cross-sectional case-control and before-after studies [18, 19]. Most of the studies (*n* = 9, 69.2%) were of ‘moderate’ quality, and two (15.4%) were of ‘low’ quality. Adequate randomisation, enough sample sizes, prespecified inclusion and exclusion criteria, specified study population, and clearly defined exposure and outcomes measures were all available in both controlled interventions [28, 31], and this increased the confidence that the reported results might have been attributable to the intervention than the difference in groups. For the before-after studies, 6 out of 9 studies had a control group [20, 23, 25, 27, 29, 32], specified inclusion and exclusion criteria, and defined exposure and outcome measures, and this also increased confidence that the reported improvements between before and after evaluations were not merely by chance. However, different participants’ selection, small sample sizes, and short follow-up periods among other studies [20–22, 28, 31], including two descriptive studies [24, 26] that did not mention how study participants were chosen or how exposure and outcomes measures were defined, might require their results to be interpreted with caution. Also, inadequate follow-up periods, failure to measure or include confounders in analyses, and lack of validity of reported outcomes might have resulted in some studies overestimated the effectiveness of the reported interventions.

## Discussion

This systematic review aimed to synthesise available evidence on treatment modalities for both cervical neoplasia and cervical cancer in HIV-seropositive women in developing countries. Most cervical cancer patients are reported to be diagnosed at an advanced stage of the disease because of the lack of coordinated and systematic screening [9, 10]. Besides, lack of optimal treatment regimen due to factors such as lack of infrastructure, financial, and human resources has been found to contribute to poor outcomes of treatment among HIV-seropositive women in developing countries [10, 13]. The findings of this systematic review have shown that the available cervical cancer treatments, radiotherapy, chemotherapy, chemoradiation, and surgery appear to be effective for HIV-seropositive patients and are the same treatments being used for HIV-negative patients as well as in developed countries. This review has also shown that opportunities to improve cervical neoplasia and cervical cancer management in HIV-positive women exist. However, developing countries need to prioritise early diagnosis and treatment of precancerous lesions to reduce cervical cancer and align with 2030 Sustainable Development Goals to reduce non-communicable diseases mortality. As most developing countries put plans and measures in place for universal health coverage by 2030, it is paramount that benefits packages to be offered should include cervical cancer screening, HPV vaccination, testing, and treatment especially for HIV-positive women if they are to achieve the same impact as developed countries.

The introduction of life-long antiretroviral (ART) has been found to moderately reduce HPV infection incidences [33]. Despite the moderate effect on HPV infection, ART is prolonging the life span of those infected with HIV, thereby granting time for the development of cervical neoplasia and cervical cancer especially in countries with not well-established cervical cancer screening programs. This systematic review has confirmed that the available treatments for both cervical neoplasia and cervical cancer (if detected early) among HIV-seropositive women appear to be effective. However, clinical, methodological, and statistical heterogeneity, such as participants’ baseline characteristics, immunosuppressive status, follow-up time, randomisation versus non-randomisation, sample sizes, and statistical calculations, among the 10 studies, might explain some the differences in the findings. In this review, almost all the included studies had HIV-seropositive women who were younger than HIV-negative women were.

This systematic review demonstrated that LEEP and cryotherapy treatments have the possibility of reducing progression from LSIL to HSIL as well as causing regression of cervical neoplasia [27, 29, 31, 32]. However, this treatment benefit was exclusively significant among women with high-risk HPV and might point to a need for further multicentre research to explore the reasons for such a finding.

In as much as LEEP was reported to be safe, several complications, such as severe intraoperative haemorrhage, early and late postoperative haemorrhage, localised infection of the cervix, and cervical stenosis, were experienced in both HIV-positive and HIV-negative women although the difference was insignificant [27]. Despite no difference in complications between HIV-positive and HIV-negative women, further research on reasons for such complications need to be assessed and explored to inform best clinical practices.

In India, treatment with radiotherapy was seen to be effective among HIV-seropositive women with cervical cancer stage IIIB-IVA [21], and these findings were supported by evidence from Kenya [22]. However, the associated acute treatment toxicity of radiotherapy among HIV-positive women was seen to be an independent significant risk factor that interrupts or delay treatment resulting in most of these women not completing their prescribed treatments [22]. Acute gastrointestinal, skin, and genitourinary tract toxicity is the most prominent radiation-related acute toxicities and is associated with HIV [21, 22]. These multisystem acute toxicity findings contrast with what was identified in a radical chemoradiation prospective study which reported no statistically significant differences between HIV-positive and HIV-negative patients [23]. Therefore, further studies examining patients’ baseline characteristics such as time of HAART or CD4+ counts will need to be conducted to analyse why studies are reporting different findings.

Being HIV-seropositive prevents the success of radiotherapy as most patients will not complete prescribed treatment due to associated multisystem toxicities hence resulting in poor response and outcomes in some cases. After 7 months post-radiotherapy, HIV-seropositive women were 3.1 times likely to have a residual tumour as compared to HIV-negative [22]. These findings indicate that completing radiation is a predictor of treatment response among HIV-seropositive women [20, 34]. Palliative radiotherapy fractionation has been reported to be effective in HIV-seropositive patients with poor performance and advanced cancer [22], but having an intact immune system and a higher CD4+ count is a positive indicator to treatment response and reduction of tumour [25].

Despite completing prescribed treatment being an indicator of treatment response in radiotherapy [21, 22], evidence on chemotherapy indicates that treatment completion did not have a greater effect or impact on the response after 6 weeks as compared to radiotherapy [20]. Besides, cervical cancer stage IIIB was indicated to be associated with poor chemo-radiation after 6 weeks [20], and this might suggest that offering a full dose of radiation coupled with good medical care in terms of associated toxicities [21, 22] might be beneficial to HIV-seropositive with advanced cervical cancer. This suggestion is supported by findings that show that chemo-radiation incremental benefit as compared to radiotherapy is minimal [35]. However, these findings required further studies to be conducted with large numbers of patients to assess the reported treatment outcomes because the evidence in Zambia has indicated that conventional doses of radical chemo-radiation are well tolerated and effective for HIV-seropositive women who are on HAART [23].

Three radical hysterectomies on reasonably stable immunosuppressive HIV-seropositive patients with cervical cancer stage IB-IIA appeared to produce better treatment and survival outcomes, with all three patients having negative vault cytologic smears after 3- and 6-years post-surgery [26]. However, because of the few patients reported in this radical hysterectomy study, there might be a need to explore further the impact of this treatment and associated outcomes.

## Limitations

Despite the overall quality of the included studies being moderate, some of the reported results were affected by the risk of bias associated with the comparability of effects, populations, and information including lack of explanations on the conducted statistical analyses. By limiting our study searches to those reported in English, this systematic review might have missed some relevant studies published in other languages.

## Conclusions

Those infected with HIV were younger and have advanced disease as compared to those who were HIV-negative [20–25, 27–29, 31, 32]. In as much as the mass HPV vaccination is targeting 9- to 13-year-old young girls, it can be argued, based on the findings from this review, that developing countries must offer targeted vaccination [36] to HIV-positive adolescents and young women between the ages of 13 and 26 years through already established HIV clinics, to increase vaccination coverage and consolidated potential benefits. This is in line with the Guidelines for Prevention and Treatment of Opportunistic Infections in HIV-Infected Adults and Adolescents [37]. Offering routine cervical cancer screening and HPV testing in HIV clinics might help in early identification of these at high-risk women who are relatively ignorant and lack knowledge about cervical cancer risk factors. Facilitation and putting HIV-infected people on life-long ART is of importance and has been found to have a positive impact on cervical cancer treatment response.

## Implications of the review’s results to evidence-based health care

Based on this review, the following key messages on the reliability of the findings have emerged:

- Both cervical neoplasia and cervical cancer in HIV-seropositive women are treatable with the available treatment, and better outcomes are associated with early diagnosis and treatment availability. Also, these are the same treatments that are available in developed countries and have made a tremendous impact. However, the differences between developing and developed countries are around lack of optimal treatment regimen and underutilisation of available cervical cancer services due to cost, lack of knowledge, lack of infrastructure, and human resources that continue to hamper developing countries. There is a need for good clinical management of HIV-seropositive women undergoing chemo-radiation to manage multisystem toxicities that have a bearing on treatment completion, prognostic, and survival outcomes. Research on cervical cancer management of HIV-seropositive patients focusing on the quality of life of those treated, the effectiveness of the treatment method considering CD4+ count, and ART is required.
- As HPV infections continue to be high among HIV-positive women regardless of ART, primary prevention through HPV vaccination is critically needed among young HIV-positive girls at the recommended ages since the vaccine is safe and beneficial to them. Most HIV-seropositive women with cervical cancer are young, and screening from the age of 15 years, taking into consideration early sexual debut and high HIV incidence, might increase early identification of at-risk young women. There is a need to strengthen health systems by establishing robust and regular cervical cancer screening and HPV testing beginning at age 21 in HIV testing and treatment clinics. Multicentre research on early screening of young women is required to inform feasibility, appropriateness, meaningfulness, and cost-effectiveness.

## Supplementary information

**Additional file 1.** PubMed and OvidSP (MEDLINE and Embase) Search Strategies. This file contains two examples of the search strategies used to search for studies that were included in this literature review. The search strategies are for PubMed, MEDLINE and Embase databases.

## Data Availability

The analysed data and materials are included in this publication.

## Abbreviation

CINAHL: Cumulative Index to Nursing and Allied Health Literature
CIN2+: Cervical intraepithelial neoplasia grade 2+
EMBASE: Excerpta Medica Database
HIV: Human immunodeficiency virus
HPV: Human papillomavirus
HPV DNA/mRNA: Human papillomavirus deoxyribonucleic acid/messenger RNA
HSIL: High-grade squamous intraepithelial lesion
LEEP: Loop electrosurgical excision procedure
LSIL: Low-grade squamous intraepithelial lesion
PRISMA: Preferred Reporting Items for Systematic Review and Meta-Analysis
RCT: Randomised controlled trial
