# Supplementary material for: Treatment of pre- and confirmed cervical cancer in HIV-seropositive women from developing countries: a systematic review
Source: Syst Rev. 2020 Apr 10;9:79. doi: 10.1186/s13643-020-01345-2 (PMC7149877; doi:10.1186/s13643-020-01345-2)
Supplement: Supplementary file 1 — Additional file 1. PubMed and OvidSP (MEDLINE and Embase) Search Strategies. This file contains two examples of the search strategies used to search for studies that were included in this literature review. The search strategies are for PubMed, MEDLINE and Embase databases. [file 13643_2020_1345_MOESM1_ESM.pdf]

File name: Additional file 1
Title of data: OvidSP (MEDLINE and Embase) and PubMed Search Strategies

OvidSP search strategy for treatment of cervical cancer in HIV-seropositive women

Search Terms	
1.	Cervi* canc*.mp. [mp=title, abstract, full text, caption text]
2.	cervi* neoplas*.mp. [mp=title, abstract, full text, caption text]
3.	cervi* carcinom*.mp. [mp=title, abstract, full text, caption text]
4.	cervi* dysplas*.mp. [mp=title, abstract, full text, caption text]
5.	cervi* intraepithelial neoplas*.mp. [mp=title, abstract, full text, caption text]
6.	treat* or therap*.mp. [mp=title, abstract, full text, caption text]
7.	chemotherap* .mp. [mp=title, abstract, full text, caption text]
8.	surger*.mp. [mp=title, abstract, full text, caption text]
9.	radiation adj3 therap*.mp. [mp=title, abstract, full text, caption text]
10.	cryotherap*.mp. [mp=title, abstract, full text, caption text]
11.	HIV positive.mp. [mp=title, abstract, full text, caption text]
12.	hiv seropositiv*.mp. [mp=title, abstract, full text, caption text]
13.	hiv.mp. [mp=title, abstract, full text, caption text]
14.	developing countr*.mp. [mp=title, abstract, full text, caption text]
15.	underdeveloped countr*.mp. [mp=title, abstract, full text, caption text]
16.	low income countr*.mp. [mp=title, abstract, full text, caption text]
17.	low resource countr*.mp. [mp=title, abstract, full text, caption text]
18.	low resource setting*.mp. [mp=title, abstract, full text, caption text]
19.	developing countries.mp. [mp=title, abstract, full text, caption text]
20.	1 or 2 or 3 or 4 or 5
21.	6 or 7 or 8 or 9 or 10
22.	11 or 12 or 13
23.	14 or 15 or 16 or 17 or 18 or 19
24.	20 and 21 and 22 and 23

PubMed search strategy for treatment of cervical cancer in HIV-seropositive women

1. Cervical Neoplasm, Uterine OR Cervical Neoplasms, Uterine OR Neoplasm, Uterine Cervical OR Neoplasms, Uterine Cervical OR Uterine Cervical Neoplasm OR Neoplasms, Cervical OR Cervical Neoplasms OR Cervical Neoplasm OR Neoplasm, Cervical OR Neoplasms, Cervix OR Cervix Neoplasms OR Cervix Neoplasm OR Neoplasm, Cervix OR Cancer of the Uterine Cervix OR Cancer of the Cervix OR Cervical Cancer OR Uterine Cervical Cancer OR Cancer, Uterine Cervical OR Cancers, Uterine Cervical OR Cervical Cancer, Uterine OR Cervical Cancers, Uterine OR Uterine Cervical Cancers OR Cancer of Cervix OR Cervix Cancer OR Cancer, Cervix OR Cancers, Cervix
2. Treatment OR therapy OR chemotherapy OR surgery OR radiation OR radiotherapy OR radiation therapy OR cryotherapy OR loop electrosurgical excision procedure OR LEEP
3. hiv seropositivity OR hiv seropositivities or seropositivities, hiv OR hiv positive OR hiv or seropositivity, hiv OR AIDS positivity OR AIDS
4. Developing Countries OR Africa OR Africa, Northern OR Africa South of the Sahara OR Africa, Central OR Africa, Eastern OR Africa, Southern OR Africa, Western OR

Asia OR Asia, Central OR Asia, Southeastern OR Asia, Western OR Caribbean Region OR West Indies OR South America OR Latin America OR Central America OR Afghanistan OR Albania OR Algeria OR American Samoa OR Angola OR "Antigua and Barbuda" OR Argentina OR Armenia OR Azerbaijan OR Bahrain OR Bangladesh OR Barbados OR Benin OR Belarus OR Belize OR Bhutan OR Bolivia OR Bosnia-Herzegovina OR Botswana OR Brazil OR Bulgaria OR Burkina Faso OR Burundi OR Cambodia OR Cameroon OR Cape Verde OR Central African Republic OR Chad OR Chile OR China OR Colombia OR Comoros OR Congo OR Costa Rica OR Cote d'Ivoire OR Croatia OR Cuba OR Cyprus OR Czechoslovakia OR Czech Republic OR Slovakia OR Djibouti OR "Democratic Republic of the Congo" OR Dominica OR Dominican Republic OR East Timor OR Ecuador OR Egypt OR El Salvador OR Eritrea OR Estonia OR Ethiopia OR Fiji OR Gabon OR Gambia OR "Georgia (Republic)" OR Ghana OR Greece OR Grenada OR Guatemala OR Guinea OR Guinea-Bissau OR Guam OR Guyana OR Haiti OR Honduras OR Hungary OR India OR Indonesia OR Iran OR Iraq OR Jamaica OR Jordan OR Kazakhstan OR Kenya OR Korea OR Kosovo OR Kyrgyzstan OR Laos OR Latvia OR Lebanon OR Lesotho OR Liberia OR Libya OR Lithuania OR Macedonia OR Madagascar OR Malaysia OR Malawi OR Mali OR Malta OR Mauritania OR Mauritius OR Mexico OR Micronesia OR Middle East OR Moldova OR Mongolia OR Montenegro OR Morocco OR Mozambique OR Myanmar OR Namibia OR Nepal OR Netherlands Antilles OR New Caledonia OR Nicaragua OR Niger OR Nigeria OR Oman OR Pakistan OR Palau OR Panama OR Papua New Guinea OR Paraguay OR Peru OR Philippines OR Poland OR Portugal OR Puerto Rico OR Romania OR Russia OR "Russia (Pre-1917)" OR Rwanda OR "Saint Kitts and Nevis" OR Saint Lucia OR "Saint Vincent and the Grenadines" OR Samoa OR Saudi Arabia OR Senegal OR Serbia OR Montenegro OR Seychelles OR Sierra Leone OR Slovenia OR Sri Lanka OR Somalia OR South Africa OR Sudan OR Suriname OR Swaziland OR Syria OR Tajikistan OR Tanzania OR Thailand OR Togo OR Tonga OR "Trinidad and Tobago" OR Tunisia OR Turkey OR Turkmenistan OR Uganda OR Ukraine OR Uruguay OR USSR OR Uzbekistan OR Vanuatu OR Venezuela OR Vietnam OR Yemen OR Yugoslavia OR Zambia OR Zimbabwe
